# Antenatal care attendance and low birth weight of institutional births in sub-Saharan Africa

**DOI:** 10.1186/s12884-022-04576-4

**Published:** 2022-04-05

**Authors:** Alirah Emmanuel Weyori, Abdul-Aziz Seidu, Richard Gyan Aboagye, Francis Arthur- Holmes, Joshua Okyere, Bright Opoku Ahinkorah

**Affiliations:** 1 Institute of Development and Agricultural Economics, Leibniz Universitat Hannover, Hannover, Germany; 2 Department of Real Estate Management, Faculty of Build and Natural Environment, Takoradi Technical University, Takoradi, Ghana; 3 Centre for Gender and Advocacy, Takoradi Technical University, Takoradi, Ghana; 4 College of Public Health, Medical and Veterinary Sciences, James Cook University, Townsville, Australia; 5 Department of Family and Community Health, School of Public Health, University of Health and Allied Sciences, Hohoe, Ghana; 6 Department of Sociology and Social Policy, Lingnan University, TuenMun, 8 Castle Peak Road, Tuen Mun, Hong Kong; 7 Department of Population and Health, University of Cape Coast, Cape Coast, Ghana; 8 School of Public Health, Faculty of Health, University of Technology Sydney, Sydney, Australia

**Keywords:** Low birth weight, Institutional births, Antenatal care, Sub-Saharan African countries

## Abstract

**Background:**

Low birth weight (LBW) remains a major health problem that affects newborns worldwide. However, there has been growing evidence that antenatal care (ANC) is associated with LBW. Yet, there is a dearth of research investigating the association between ANC attendance and LBW in sub-Saharan Africa (SSA). This study examined the association between the number of ANC visits and LBW using data from 10 sub-Saharan African countries.

**Methods:**

This study pooled data from the recent Demographic and Health Survey (DHS) of 10 sub-Saharan African countries conducted from 2018 to 2020. A total of 33,585 women aged 15–49 who had live births in the five years preceding the survey were included in this study. Bivariable and multivariable multilevel regression models were fitted to show the association between the number of ANC visits and LBW. Crude odds ratio (cOR) and adjusted odds ratio (aOR) at 95% confidence intervals (CIs) were used in presenting the results of the regression analysis.

**Results:**

The pooled prevalence of LBW was 5.7%. The highest prevalence of LBW was recorded in Gambia (7.2%) with the lowest found in Sierra Leone (2.9%). In terms of eight or more ANC visits, the overall prevalence was 14.5%. Nigeria had the highest prevalence of eight or more ANC visits (43.5%) with the lowest in Rwanda (0.2%). We found a statistically significant association between the number of ANC visits and LBW. Mothers who had eight or more ANC visits were less likely to have LBW children compared to mothers who had less than eight ANC visits [cOR = 0.66; CI = 0.55 – 0.79] and this persisted after controlling for the covariates [aOR = 0.68; CI = 0.56 – 0.82]. Covariates associated with LBW were maternal age, marital status, level of education, age of child, and wealth index.

**Conclusion:**

This study has shown a statistically significant association between ANC and LBW in SSA, with women who had eight or more ANC visits being at lower risks of giving birth to children with LBW. We found that eight or more ANC attendance was a protective factor against LBW in SSA. Therefore, it is important for sub-Saharan African countries with low prevalence of eight or more ANC attendance and high LBW prevalence to channel their efforts towards promoting more ANC attendance.

## Background

 Newborn's weight has become a topical issue in public health recently with a focus on low birth weight [LBW] [1]. The World Health Organization (WHO) defines LBW as a birth weight of a live born infant that is < 2500 g irrespective of the gestational age or other predictors [2]. LBW has been recognized as a significant predictor of morbidity, mortality, and disability [3]. The heightened interest for LBW is influenced primarily by the fact that it is a perfect indicator for assessing both maternal and child health status, particularly in the perinatal stage [2, 4]. For instance, WHO [5] posits that LBW is the leading cause of neonatal mortality and a predominant predictor of childhood morbidity and mortality.

LBW of a child is a global issue affecting both high-income countries and low-and-middle countries [LMICs]; however, it is prominent in LMICs including those in sub-Saharan Africa [SSA] [6]. This may be attributed to the porous nature of the social support and weak health infrastructure in most developing countries, which results in negative health outcomes. For instance, the Demographic and Health Survey (DHS) report for Ghana [7] shows a LBW prevalence of 9.5%.

There has been a growing evidence that antenatal care [ANC] attendance is associated with LBW [2, 6, 8]. Initially, the WHO came out with the four-visit ANC model as the recommended model for ANC, however, the new model in 2016 recommends that ANC should be delivered at a minimum of eight contacts [5]. There is ample evidence showing that attending less than the recommended number of ANC visits doubled the risk of LBW [5, 9]. Thus, making ANC visits critical to child health outcomes. This viewpoint is supported by the fact that ANC provides an avenue to reach pregnant women with various interventions that improve maternal and childhealth outcomes [10]. For example, during ANC visit, pregnant women are provided with various services which may include Intermittent preventive treatment in pregnancy [IPTp] for malaria control and tetanus toxoid-diphtheria vaccination which are essential to the health of the expected mother and the unborn child [8]. Essentially, the frequency and quality of ANC as well as the adherence to ANC protocols may influence LBW.

Despite the high prevalence of LBW in LMICs, most studies on ANC and LBW have been conducted in high income countries [5]. Recognizing the socio-cultural differences that exist in ANC in different regions, the findings from such studies may not be applicable to the sub-Saharan African context. Moreover, earlier studies conducted to investigate the association between ANC and LBW were based on the a minimum of four ANC contacts [2, 6, 8]. However, this study examines whether there is an  association between eight or more ANC attendance and LBW in SSA. The results from this study will be helpful in informing the design and development of policies and interventions that are best suited to countries in SSA. The study will also help relevant authorities to intensify efforts towards the improvement in ANC attendance and reduction in LBW in SSA.

## Methods

### Data source

This study pooled data from the DHS of 10 sub-Saharan African countries (Table 1). The DHS is a nationally representative survey that is conducted every 5 years in over 85 LMICs. The DHS collects data in several health and social indicators including maternal and child health makers such as child’s size at birth and ANC attendance [11]. DHS employed a cross-sectional design and this design was carried out descriptively among the respondents in the surveyed countries. The design enables for the measurement of outcome and exposure variables simultaneously among a subset of the population of interest. The study population for the DHS survey consist of men, women, and children. However, making this study, the data was pooled from the kid’s recode (KR) files, which contain information on live births to interviewed women born 5 years prior to the survey. In addition, only countries with dataset, which span from 2018 to 2020, were considered for the analysis. This is because; only these countries had data, which were collected after the introduction of the new ANC contact model by the WHO [5]. The DHS employs a two-stage stratified sampling technique. In the first stage, clusters are selected using a probability proportional to size (PPS) sampling technique. In the second stage, a predetermined number of households (usually 28–30) are selected using a systematic sampling technique. The study by Aliaga and Ruilin [12] provides details of the sampling process. In this study, 33,585 women aged 15–49 who had live births in the five years preceding the survey were included. We relied on the Strengthening the Reporting of Observational Studies in Epidemiology’ (STROBE) statement in writing the manuscript [13]. The dataset is freely available for download at: https://dhsprogram.com/data/available-datasets.cfm.

**Table 1:** Description of the study sample

Country	Year of survey	Weighted N	Weighted %
1. Benin	2018	7,058	21.0
2. Cameroon	2018	2,177	6.5
3. Gambia	2019–20	2,200	6.5
4. Guinea	2018	1,279	3.8
5. Liberia	2019–20	1,519	4.5
6. Mali	2018	4,244	12.6
7. Nigeria	2018	3,659	10.9
8. Rwanda	2019–20	2,807	8.4
9. Sierra Leone	2019	2,844	8.5
10. Zambia	2018	5,798	17.3
All countries		**33,585**	**100.0**

### Selection of variables and measurement

#### Outcome variable

The outcome variable for this study was LBW. It was obtained from the birth weight data which was recorded from mothers whose youngest child was less than five years old in the five years prior to the survey using health card records. For entries on health cards, physician or a health worker completed them and then gave to mothers upon their discharge from the health facility (e.g., hospital, clinic or any other healthcare institution) [10, 14]. It is stated that reporting birth weight information on health cards is more reliable than birth weight information obtained through maternal recall [15–17]. Birth weight data were classified into two groups: non-LBW (birth weight ≥ 2500 g) or LBW (birth weight < 2500 g). Very crucially, data on children with a missing birth weight, mothers with twin or multiple pregnancies, stillbirths and children who were not born in health facilities were excluded from the analysis.

#### Key explanatory variable

The main explanatory variable of this study was “number of ANC visits”. To derive this variable, the DHS asked a question “How many times did you receive ANC during this pregnancy? The responses to this question were categorized as < 8 visits or ≥ 8 visits. This categorization was in line with the WHO recommended eight-visit ANC model which was developed in 2016 [5].

#### Covariates

Nine control variables were considered in this study. These included maternal age (15–19, 20–24, 25–29, 30–34, 35–39, 40–44, 45–49), marital status (currently married, cohabiting, previously married), pregnancy intention (intended, mistimed, unwanted), place of residence (urban, rural), mother’s educational level (no education, primary, secondary/higher), wealth index (poorest, poorer, middle, richer, richest), child’s age (0–11 months, 24–35 months, 36–47 months, 48–59 months), sex of child (male and female), and age at first childbirth (< 20 years and 20 years and above).

#### Data analyses

**Fig. 1:**
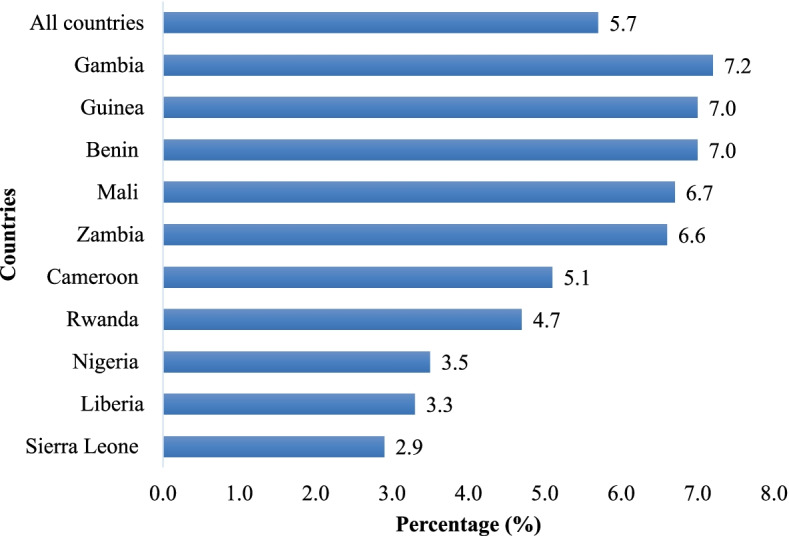
Prevalence of low birth weight

**Fig. 2:**
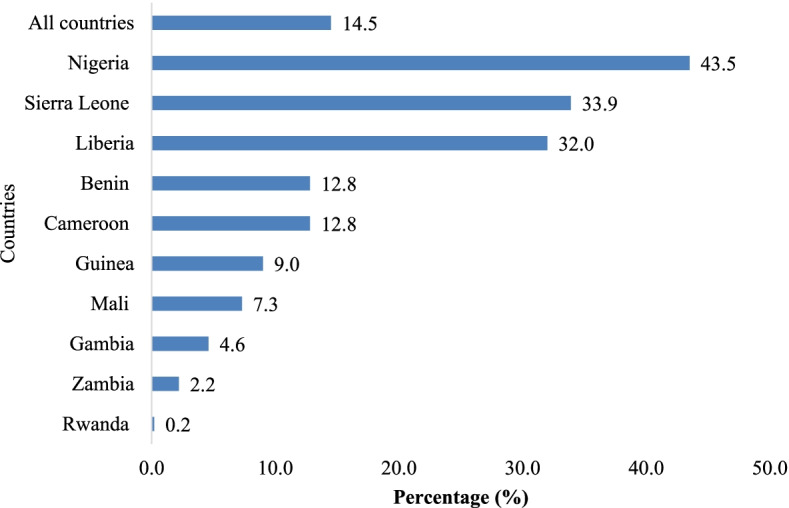
Prevalence of eight or more ANC attendance

Data analyses were carried out using Stata version 16.0. The analyses were conducted in four steps. The first step was a graphical representation of the prevalence of LBW (Fig. 1) and the prevalence of eight or more ANC visits in SSA (Fig. 2). The second step was a bivariate analysis that calculated the proportion of LBW across the explanatory variable with their *p*-values generated from a Chi-square test analysis (Table 2). For the third step, a four modelled multilevel logistic regression was used to examine the association between eight or more ANC visits and LBW (Table 3). The first model (Model I) was a null model with no explanatory variables or covariates, and it demonstrated variance in LBW attributed to the primary sampling units (PSU). The second model, Model II, included only the key explanatory variable (eight or more ANC visits), while the third model (Model III), included only the covariates. The final model (Model IV) included eight or more ANC visits and the covariates together. Fixed and random effects were included in Model II-IV. The fixed effects represented the relationship between the eight or more ANC visits and/or covariates and the LBW, while the random effects represented the measure of variation in the LBW based on PSU, as measured by Intra-Cluster Correlation (ICC). Finally, model fitness was evaluated using the Akaike's Information Criterion (AIC). Crude odds ratio (cOR) and adjusted odds ratio (aOR) at 95% confidence intervals (CIs) were used in presenting the results. Variance inflation factor was used to check for multicollinearity among the independent variables, and there was no evidence of collinearity. To run the multilevel regression models, we used the "mlogit" command. Weighting was used to account for the complexities of DHS data, and the "svyset" command was used to account for disproportionate sampling and non-response.

**Table 2:** Distribution of low birth weight across number of ANC visits and the sociodemographic characteristics of the respondents

Variables	Weighted %	Weighted %	LBW	*P*-value
**ANC visits**				< 0.001
Less than 8	28,702	85.5	6.0	
Eight or more	4,883	14.5	4.0	
**Maternal age**				< 0.001
15–19	2,451	7.3	9.5	
20–24	7,120	21.2	7.0	
25–29	8,600	25.6	5.3	
30–34	7,006	20.9	4.6	
35–39	5,390	16.0	4.7	
40–44	2,293	6.8	5.8	
45–49	725	2.2	3.3	
**Marital status**				0.006
Single	4,522	13.5	6.6	
Married	25,341	75.4	5.7	
Cohabiting	3,722	11.1	4.7	
**Maternal education**				0.570
No education	12,178	36.3	5.9	
Primary	9,236	27.5	5.7	
Secondary or higher	12,171	36.2	5.5	
**Pregnancy intention**				0.018
Then	24,524	73.0	5.6	
Later	7,176	21.4	6.2	
No more	1,885	5.6	4.3	
**Child’s age (months)**				0.015
0–11	10,397	31.0	6.0	
12–23	9,342	27.8	5.4	
24–35	6,652	19.8	6.4	
36–47	4,349	12.9	5.0	
48–59	2,844	8.5	4.9	
**Child’s sex**				< 0.001
Male	17,196	51.2	5.0	
Female	16,389	48.8	6.4	
**Age at first birth**				0.048
Adolescent birth	17,977	53.5	6.0	
Adult birth	15,608	46.5	5.4	
**Wealth index**				0.017
Poorest	5,269	15.7	5.1	
Poorer	6,224	18.5	5.9	
Middle	6,748	20.1	6.6	
Richer	7,658	22.8	5.4	
Richest	7,685	22.9	5.4	
**Residence**				0.963
Urban	14,773	44.0	5.7	
Rural	18,812	56.0	5.7	

^*^P-values were generated from the Pearson chi-square test

**Table 3:** Mixed effects analysis of association between antenatal attendance and low birth weight

Variables	Model I	Model II cOR [95% CI]	Model III aOR [95% CI]	Model IV aOR [95% CI]
***Fixed effect results***				
**ANC visits**				
Less than 8		1.00		1.00
Eight or more		0.66^***^ [0.55—0.79]		0.68^***^ [0.56—0.82]
**Maternal age**				
15–19			1.00	1.00
20–24			0.69^***^ [0.57—0.84]	0.69^***^ [0.57—0.83]
25–29			0.50^***^ [0.41—0.62]	0.50^***^ [0.41—0.62]
30–34			0.43^***^ [0.35—0.54]	0.44^***^ [0.35—0.55]
35–39			0.43^***^ [0.34—0.55]	0.44^***^ [0.34—0.56]
40–44			0.55^***^ [0.42—0.73]	0.55^***^ [0.42—0.74]
45–49			0.30^***^ [0.19—0.48]	0.30^***^ [0.19—0.48]
**Marital status**				
Married			1.00	1.00
Cohabiting			0.81^*^ [0.66—0.98]	0.82^*^ [0.68—1.00]
Single			1.03 [0.87—1.22]	1.03 [0.87—1.22]
**Maternal education**				
No education			1.00	1.00
Primary			0.90 [0.78—1.04]	0.90 [0.78—1.03]
Secondary or higher			0.83^*^ [0.72—0.96]	0.84^*^ [0.73—0.98]
**Age at first birth**				
Adolescent birth			1.00	1.00
Adult birth			1.11 [0.98—1.25]	1.12 [0.99 -1.26]
**Pregnancy intention**				
Then			1.00	1.00
Later			1.03 [0.90—1.18]	1.01 [0.88—1.16]
No more			0.85 [0.66—1.11]	0.85 [0.65—1.10]
**Child’s age (months)**				
0–11			1.00	1.00
12–23			0.94 [0.82—1.07]	0.94 [0.82,1.08]
24–35			1.17^*^ [1.02—1.36]	1.18^*^ [1.02,1.36]
36–47			0.95 [0.78—1.15]	0.95 [0.79,1.15]
48–59			0.96 [0.76—1.22]	0.96 [0.76,1.22]
**Child’s sex**				
Male			1.00	1.00
Female			1.31^***^ [1.18—1.45]	1.31^***^ [1.18—1.45]
**Wealth index**				
Poorest			1.00	1.00
Poorer			1.17 [0.99—1.40]	1.17 [0.99—1.40]
Middle			1.33^**^ [1.10—1.60]	1.33^**^ [1.11—1.60]
Richer			1.07 [0.88—1.30]	1.08 [0.89—1.31]
Richest			1.14 [0.92—1.41]	1.17 [0.95—1.45]
**Residence**				
Urban			1.00	1.00
Rural			0.94 [0.82—1.07]	0.92 [0.80—1.06]
***Random effect model***				
PSU variance (95% CI)	0.119 [0.072–0.196]	0.115 [0.069–0.192]	0.111 [0.065–0.189]	0.108 [0.063–0.188]
ICC	0.035	0.034	0.033	0.032
Wald chi-square	Reference	20.15 (< 0.001)	176.70 (< 0.001)	193.35 (< 0.001)
**Model fitness**				
Log-likelihood	-7405.9741	-7389.9778	-7308.1333	-7294.9571
AIC	14,815.95	14,785.96	14,666.27	14,641.91
N	33,585	33,585	33,585	33,585
Number of clusters	1,253	1,253	1,253	1,253

*aOR* adjusted odds ratios, *CI* Confidence Interval, *cOR* Crude odds ratio; ^*^ *p* < 0.05, ^**^ *p* < 0.01, ^***^ *p* < 0.001; 1 = Reference category; *PSU* Primary Sampling Unit, *ICC* Intra-Class Correlation, *AIC* Akaike’s Information Criterion

## Results

### Prevalence of eight or more ANC visits and low birth weight in sub-Saharan Africa

For the ten sub-Saharan African countries considered in this study, the prevalence of LBW was 5.7%. The highest prevalence of LBW was recorded in Gambia (7.2%) with the lowest found in Sierra Leone (2.9%) (Fig. 1). In terms of eight or more ANC visits, the overall prevalence was 14.5%. Nigeria had the highest prevalence of eight or more ANC visits (43.5%) with the lowest in Rwanda (0.2%) (Fig. 2).

### Distribution of low birth weight by the socio-demographic characteristics of respondents

Table 2 shows results of the distribution of LBW by the socio-demographic characteristics of the study respondents. The Chi-square test results showed significant variations in LBW across number of ANC visits, maternal age, marital status, pregnancy intention, child’s sex, child's age, age at first birth, and wealth index. Specifically, the highest prevalence of LBW was found among women who had less than eight ANC visits (6.0%), those aged 15–19 (9.5%), those whose pregnancy intention was later (6.2%), female children (6.4%), mothers with no education (5.9%), mothers who belonged to the middle wealth index (6.6%), and those whose first birth occurred when they were adolescents  (6.0%).

### Association between number of ANC visits and low birth weight in sub-Saharan Africa

Table 3 presents results on the association between number of ANC visits and LBW. We found a statistically significant association between the number of ANC visits and LBW. Mothers who had eight or more ANC visits were less likely to have LBW children compared to mothers who had less than eight ANC visits [cOR = 0.66; CI = 0.55 – 0.79] and this persisted after controlling for the covariates [aOR = 0.68; CI = 0.56 – 0.82]. Covariates associated with LBW were maternal age, marital status, level of education, age of child, and wealth index.

## Discussion

LBW among children is a serious public health concern that contributes to child morbidity and mortality worldwide [18]. We investigated the association between ANC attendance and child birth weight within the sub-Saharan African context. Our findings shows an overall LBW prevalence of 5.7% across the ten countries included in this study. The observed prevalence of LBW is lower than what has been reported in an earlier study [19] that found a prevalence of 9.7%. Probably, the difference between our obeserved prevalence of LBW and that of Tessema et al.’s [19] could be due to the differences in the number of countries included in the analysis. In Tessema et al.’s [19] study, 35 countries in SSA were included whereas in this study, we included only ten countries in SSA. Hence, accounting for the observed low prevalence of LBW. Notwithstanding, the study shows that Gambia reported the highest prevalence of LBW while Sierra Leone reported the lowest prevalence. It is uncertain what may be accounting for the differences in LBW across sub-Saharan African countries. However, Sierra Leone’s low prevalence of LBW could be due to investments such as the ‘Free Health Care’ scheme which promotes community-based packages of care to improve linkage and referral for facility births [20].

We found the pooled prevalence of eight or more ANC visits to be very low (14.5%). The WHO recommends that women receive a minimum of eight ANC visits to optimise both maternal and child health outcomes [5]. Therefore, the low prevalence of eight or more ANC visits as reported in this study, is a threat to the attainment of the sustainable development goals (SDGs), especially target 3.1 and 3.2 [21]. This finding underscores the need for sub-Saharan African countries to reassess their commitments and interventions channelled towards improving ANC attendance. In our study, Nigeria had the highest prevalence of eight or more ANC visits while Rwanda had the least prevalence. A plausible explanation for this variation in the prevalence of ANC attendance between sub-Saharan countries could be the fact that different countries may have different benchmarks for ANC. Therefore, while some countries use the WHO’s latest recommendation of eight ANC contacts as a minimum, others may still operate with four ANC contacts as a minimum. For instance, since 2003, Rwanda implemented the focused antenatal care (FANC) strategy which encourages women to have four ANC visits [22]. Unlike Nigeria where ANC is free in about 18 of its 36 states [23], pregnant women in Rwanda have to pay for ANC visits through the co-payment system [22]. Such out-of-pocket-payments in Rwanda might serve as barrier for women to attain eight or more ANC contact unless in cases of complications.

We found a significant association between number of ANC visits and LBW, with mothers who had eight or more ANC visits less likely to have LBW children compared to mothers who had less than eight ANC visits. This persisted after controlling for the covariates (maternal age, marital status, pregnancy intention, child’s sex and maternal BMI). This finding is consistent with that of Banchani and Tenkorang [24] which demonstrates that women who completed the recommended eight ANC contacts had a significantly reduced likelihood of giving birth to a child with LBW. The finding is further supported by Zhou et al. [6] who found that women with less than eight ANC visits were more likely to give birth to children with LBW. Similarly, the finding also supports that of Appiah et al. [8] showing that women who attended ANC sessions less than the recommended number had a lower likelihood of having a child with LBW. The findings could be due to the fact that the more women attend ANC sessions, the more likely they would gain maternal and child health knowledge, and apply it. Another reason could be that during the ANC session, possible risk factors of LBW are screened for and subsequently, requisite preventive measures are carried out [25]. This helps to reduce the odds of giving birth to a child with LBW.

### Strength and limitations

The strength of this study lies in the use of a nationally representative dataset from 10 sub-Saharan African countries. This provides the study with statistial power to generalize the results to women in the selected sub-Saharan African countries in this study. Despite these strengths, the study has some limitations. A major limitation of the study is that the data used employed cross-sectional design. Hence, we cannot establish causal inferences between the explanatory and outcome variables. Also, the study is limited only to women between 15–49 years in the countries that were included in the study. Due to the use of a secondary data source, the variables analyzed were restricted to only those in the data set. Therefore, important factors (e.g. beliefs about ANC) that could influence the association between ANC and LBW could not be examined. Lastly, the ANC visit was self-reported and was prone to recall and social desirability biases.

## Conclusion

 The study has shown a statistically significant association between ANC and LBW in SSA, with women who had eight or more ANC sessions being at lower risks of giving birth to a child with LBW. Therefore, it is important for sub-Saharan African countries with low prevalence of eight or more ANC attendance and high LBW prevalence to channel their efforts towards promoting more ANC attendance. Since this study did not consider the quality of ANC, we recommend that future studies collect primary data on quality ANC and explore its association with LBW.

## Data Availability

The dataset is freely available for download at: https://dhsprogram.com/data/dataset/Benin_Standard-DHS_2017.cfm?flag=1 https://dhsprogram.com/data/dataset/Cameroon_Standard-DHS_2018.cfm?flag=1 https://dhsprogram.com/data/dataset/Gambia_Standard-DHS_2019.cfm?flag=1 https://dhsprogram.com/data/dataset/Guinea_Standard-DHS_2018.cfm?flag=1 https://dhsprogram.com/data/dataset/Liberia_Standard-DHS_2019.cfm?flag=1 https://dhsprogram.com/data/dataset/Mali_Standard-DHS_2018.cfm?flag=1 https://dhsprogram.com/data/dataset/Nigeria_Standard-DHS_2018.cfm?flag=1 https://dhsprogram.com/data/dataset/Rwanda_Standard-DHS_2019.cfm?flag=1 https://dhsprogram.com/data/dataset/Sierra-Leone_Standard-DHS_2019.cfm?flag=1 https://dhsprogram.com/data/dataset/Zambia_Standard-DHS_2018.cfm?flag=1

## Abbreviations

LBW: Low birth weight
ANC: Antenatal care
DHS: Demographic and Health Survey
cOR: Crude Odds Ratio
aOR: Adjusted Odds Ratio
CI: Confidence intervals
WHO: World Health Organization
